# Thermal persistence and physicochemical modification of inorganic gunshot residue (IGSR)

**DOI:** 10.1111/1556-4029.70434

**Published:** 2026-08-10

**Authors:** Gianmarco Argentiero, Barbara Bertoglio, Nicolas Martinelli, Giulia Pezzoni, Giorgio Marrubini, Federica Maraschi, Giovanni Cecchetto, Silvia D. Visonà, Daniele Merli

**Affiliations:** ^1^ Unit of Forensic Medicine and Forensic Sciences, Department of Public Health, Experimental and Forensic Medicine University of Pavia Pavia Italy; ^2^ Department of Drug Sciences University of Pavia Pavia Italy; ^3^ Department of Chemistry University of Pavia Pavia Italy

**Keywords:** burned remains, forensic ballistics, gunshot residue (GSR), inductively coupled plasma optical emission spectroscopy, scanning electron microscopy (SEM), thermal degradation

## Abstract

In the medicolegal context, GSR analysis plays a key role in clarifying the dynamics of firearm‐related deaths, including lesion identification and firing distance determination. However, postmortem alterations of the body, such as thermal exposure, may influence the analysis of lesions and complicate interpretation. This study aimed to evaluate the persistence and modifications of GSR under different firing distances and thermal treatments using Scanning Electron Microscopy equipped with Energy‐Dispersive X‐ray Spectroscopy (SEM‐EDS) and Inductively Coupled Plasma Optical Emission Spectroscopy (ICP‐OES). Ballistic tests were conducted on calcium–magnesium silicate substrates using a self‐reloading 9 × 21 mm pistol at five barrel‐to‐target distances (5–100 cm). To simulate fire exposure, a subset of the targets was heat‐treated in a muffle furnace for 30 min at 250, 750, and 1000°C. SEM‐EDS and ICP‐OES analyses were then performed, focusing on morphology and chemical composition. GSR remained detectable under all experimental conditions. Both firing distance and thermal exposure affected gunshot residues, leading to changes in morphology, particle size, and elemental composition. An antagonistic interaction between these variables on GSR composition was identified, indicating that, depending on the temperature exposure, metal concentrations may vary with firing distance. Among the metallic elements, lead showed the strongest response, as explained by its low melting point. Notably, at 1000°C, no particles fulfilled the ASTM E1588‐25 criterion for characteristic GSR (Pb, Ba, and Sb), rendering the trimetallic population undetectable. These results highlight changes in GSR characteristics compared to standard criteria, suggesting the need to adapt GSR protocols in cases of burned remains.

**Highlights**

- Thermal exposure induces marked changes in GSR morphology, size, and elemental composition.
- At 1000°C, characteristic GSR (Pb‐Ba‐Sb) is absent; IGSR persists as bi‐ or monometallic Ba particles.
- Lead exhibits the greatest thermal sensitivity among GSR elements due to its low melting point.

## 1 INTRODUCTION

Gunshot residue (GSR) analysis is a well‐established tool in forensic casework, supporting the reconstruction of firearm‐related events. The detection and characterization of inorganic GSR (IGSR), originating primarily from primer components, can assist in evaluating firearm discharge‐related questions, including the possible presence of a shooting event, the nature of the ammunition, and—under certain circumstances—the firing distance and event dynamics [1]. Because IGSR interpretation relies largely on particle morphology and elemental composition, the analytical approach and the condition of the contaminated item are critical to the reliability of conclusions. The standard practice for forensic IGSR examination is ASTM E1588‐25 [2], which defines the morphological and compositional criteria for particle classification: particles containing Pb, Ba, and Sb in characteristic combination are classified as characteristic of GSR, whereas those containing only a subset of these elements, or morphologically consistent particles with other compositions, are categorized as consistent with or supportive of GSR.

A substantial body of literature has described IGSR morphology and chemistry under controlled conditions and explored the influence of variables such as ammunition type and barrel‐to‐target distance (e.g., [3, 4, 5, 6, 7]). In operational settings, however, the evidentiary substrate is frequently altered by post‐event processes. In human remains, decomposition and taphonomic changes may modify the distribution and detectability of residues, complicating interpretation. A particularly challenging scenario is the carbonized corpse. Fire scenes—including residential, vehicle, confined‐space, and outdoor fires—can expose victims and associated evidence to widely variable thermal and environmental conditions (e.g., temperature, exposure duration, proximity to flame, protective effects of clothing or objects, and fire suppression measures) [8, 9]. With increasing thermal damage, the recognition and interpretation of firearm‐related injuries becomes progressively more difficult [10, 11]. Although ballistic trauma features may remain identifiable even after severe carbonization in some cases [10, 12, 13], scene dynamics, suppression methods, and incomplete recovery can hinder reconstruction [12]. In addition, heat‐related artifacts can mimic traumatic lesions [14, 15]. Under these circumstances, IGSR examination may provide valuable complementary information to support trauma interpretation and reconstruction.

Only a limited number of studies have specifically addressed IGSR preservation following burning or carbonization of biological substrates, including animal bones and human samples, using approaches such as scanning electron microscopy coupled with energy‐dispersive X‐ray spectrometry (SEM‐EDS), inductively coupled plasma techniques (e.g., ICP‐MS), and imaging methods [1, 11, 16, 17, 18, 19]. Collectively, these reports indicate that IGSR may persist after thermal exposure and may undergo morphological and compositional changes. However, the available evidence is largely qualitative and has predominantly focused on close‐ or near‐contact firing conditions, often considering a single exposure temperature (commonly within the 600–800°C range). As a result, there is currently limited guidance on how IGSR deposited at different firing distances behaves when subjected to progressively increasing thermal exposure—an issue directly relevant to the interpretation of burned evidence, particularly in high‐temperature scenarios. While IGSR constitutes the primary forensic target in GSR analysis, the organic fraction of GSR (OGSR)—comprising nitroglycerin derivatives, nitrocellulose breakdown products, and primer‐specific organic markers [20]—has traditionally played a role in firing distance estimation, particularly at short ranges. In the context of the present study, OGSR was not evaluated for two principal reasons: organic GSR components are thermally labile and are expected to degrade or volatilize at temperatures well below those investigated here, rendering the organic fraction analytically uninformative in high‐temperature fire scenarios; and the analytical platform employed (SEM‐EDS and ICP‐OES) is designed for elemental and particulate characterization and does not address organic residues, which require dedicated chromatographic techniques (e.g., LC–MS/MS or GC–MS) [21].

Accordingly, the aim of this study was to evaluate IGSR persistence and modification on a fire‐resistant shooting substrate following discharge at multiple barrel‐to‐target distances (5, 25, 50, 75, and 100 cm) and subsequent exposure to increasing temperatures (250, 750, and 1000°C). Residues were characterized using SEM‐EDS (particle morphology and elemental composition, consistent with ASTM E1588‐25 [2]) and ICP‐OES (bulk elemental trends). The study was designed to (I) assess IGSR detectability after thermal exposure across distances, (II) describe temperature‐ and distance‐associated changes in particle morphology and size, and (III) evaluate compositional trends relevant to IGSR interpretation in fire‐affected casework.

## 2 MATERIALS AND METHODS

### 2.1 Target preparation and ballistic test

Shooting targets were prepared by assembling 21 × 21 cm heat‐fireproof sheets of calcium–magnesium silicate (thickness 2 mm, density 210 kg/m^3^, thermal stability >1200°C, tensile strength >0.45 MPa, shrinkage <2%, purchased from RS components, item number 724–8906) on a Whatman® Grade 42 ashless filter paper and a cardboard of equal size [6]. The target was chosen due to its fireproof characteristic, its ability to retain GSR due to the tissue‐like morphology, and the similarity to common indument tissue, thus representing a good simulation of a real‐case scenario.

The ballistics tests were performed at a Firing Ground by a single shooter, using a self‐reloading pistol, model 98FS, Cal. 9/21 mm, by Beretta (Fabbrica d'Armi Pietro Beretta, Gardone V.T., Italy) and Geco FMJ 9 mm ammunition (SINOXID primer [6]). Five different barrel‐to‐target distances were considered: 5, 25, 50, 75, and 100 cm (Figure S1). This range was selected to represent close‐ to intermediate‐range discharge scenarios, within which GSR surface density remains proportional to firing distance. For each distance, 6 different replicates were performed, for a total of 30 rounds, by shooting each target with a single projectile. Each target was then separately sealed in a plastic envelope to avoid any possible contamination.

Negative control samples (field blank) were carried out by exposing three targets in the shooting range, 1 meter behind the shooter [22].

### 2.2 Sample preparation

The targets were cut into four 7 × 7 cm quadrants, sharing a vertex in the bullet hole, and numbered from I to IV clockwise [6]. This procedure yielded four adjacent sections centred on the bullet entry point, covering a 14 × 14 cm central area of the target; the peripheral border of approximately 3.5 cm on each side was excluded from analysis, as it falls outside the primary GSR deposition zone [5, 6, 7]. From each panel, two opposite quadrants (i.e., quadrants I and III) were selected and heat‐treated in a muffle furnace for 30 min at one of the following temperatures: 250 ± 2, 750 ± 5, or 1000 ± 5°C in air (Figure S2). These temperatures were selected to represent distinct phases of thermal exposure encountered in forensic fire scenarios: 250°C corresponds to low‐temperature smoldering conditions at which organic matter begins to char while inorganic components may remain largely unaltered; 750°C represents conditions associated with established structural fires, within the range commonly reported in studies of burned bone and cremation; 1000°C approximates the upper boundary of extreme thermal exposure, as encountered in intense localized fires when the use of accelerants is of concern and in professional cremation furnaces. The remaining two quadrants (i.e., quadrants II and IV) served as unheated controls (stored at room temperature, 20–25°C). Two replicates were heat‐treated at each temperature for each distance, for a total of four burned and four unburned quadrants.

### 2.3 Inductively coupled plasma optical emission spectroscopy (ICP‐OES) analysis

ICP‐OES analysis was carried out on heat‐treated samples, considering all temperatures (including room temperature) and distances. Four field blanks and four quadrants collected from the fireproof sheet (target blank) were also included as controls to assess the presence of environmentally dispersed elements deposited on the tissues, as well as elements originally present in the target material.

Samples were treated according to a previously published procedure [7]. Briefly, the 7× 7 cm quadrants were cut into strips approximately 1 cm wide and incubated in a 50.0 mL Falcon polypropylene tube with 10 mL of 5% nitric acid (from ultrapure 65% w/w HNO₃, Merck KGaA, Darmstadt, Germany, diluted in purified water produced by a Milli‐Q Direct system by Merck Millipore, Burlington, Massachusetts, USA) for 20 h. The samples were then sonicated in an ultrasonic bath for 20 min, diluted with 10 mL of distilled water, and, after removing the strips, the extraction solution was centrifuged at 3500 rpm for 15 min and filtered through a 0.22 μm Nylon syringe filter (diameter 2 cm, Whatmann, Cytiva, Marlborough, USA). ICP‐OES analysis was carried out in an iCAP 7400 Duo (Thermo Fisher Scientific Inc., Waltham, USA), equipped with a concentric nebulizer, a cyclonic spray chamber, and a ceramic duo‐torch, according to manufacturer instructions. An ASX‐560 autosampler (Teledyne CETAC, Omaha, Nebraska, USA) was used to transfer samples in the system and data acquisition was performed using the Thermo Scientific Qtegra™ software v. 2.8.2944.202. Elements characteristic of gunshot residue (GSR) were selected, including lead, barium, and antimony for the primer mixture, as well as copper and zinc for the bullet jacket. Calibration curves were drawn by using ICP‐grade stock standard elements solutions (1000 mg/L in 1% nitric acid or hydrochloric acid (Sb), Merck KGaA, Darmstadt, Germany) at concentrations of 0.1, 1, 5 and 10 mg/L. The solutions were acidified with ultrapure nitric acid (65% w/w) to a final concentration of 2.5% w/w, ensuring compatibility with the expected analyte concentrations. Method quantification limit (MQL), expressed in μg/cm^2^ of blank tissue, was calculated from the instrumental quantification limit (LOQ) by considering the entire extraction process. The LOQ was computed as μ + 10σ, where μ corresponds to the mean concentration of the element in the 2.5% w/w nitric acid solution, and σ represents the standard deviation. Detailed data on ICP‐OES wavelengths, measurement modes, as well as detection and quantification limits (LOD/LOQ, MDL/MQL), can be found in Tables S1 and S2. Data presented are the mean of 2 quadrants from 2 independent replicates [6].

### 2.4 Scanning electron microscopy (SEM) analysis

SEM analysis was performed on nine heated samples: five samples heat‐treated at 250°C (one for each shooting distance) and four heat‐treated at 750 and 1000°C (two for each temperature, at 5 and 75 cm distances). Extending the analysis to all five distances under these conditions was therefore considered unlikely to yield sufficient particle counts for meaningful morphological and compositional characterization at the longer range endpoints. In addition, an untreated sample was included for each distance.

Inorganic GSR particles were collected by dabbing the target surface around the bullet hole area until the adhesion was lost, using a half‐inch pin‐type aluminum stub, coated with double‐sided adhesive carbon tape.

The analyses were carried out using the scanning electron microscope EM30X (COXEM), equipped with an EDS microprobe (Oxford), using an accelerating voltage of 20 keV. IGSR particles were detected by their backscattered electron signal and were described based on morphology (i.e., spherical, elliptical, and irregular) and dimension (<1 μm; 1 to 5 μm; 5 to 10 μm and >10 μm), using secondary and backscattered electrons. Particles were classified as spherical when exhibiting a rounded, quasi‐circular profile with smooth surfaces and aspect ratio approaching 1:1; as elliptical when presenting an elongated profile with smooth, well‐defined contours and aspect ratio greater than approximately 1.5:1; and as irregular when exhibiting non‐geometric profiles, including angular, fragmented, or substrate‐associated forms [23, 24]. Classification was performed by one primary examiner; ambiguous cases were reviewed by a second examiner, and discordant assignments resolved by consensus. Elemental composition was defined by acquiring the EDS spectra, focusing on the following elements: barium (Ba), lead (Pb), antimony (Sb), copper (Cu), and zinc (Zn). The SEM‐EDS review was not restricted to characteristic GSR particles alone: all particles detected by backscattered electron signal and containing at least one of the five target elements were included, regardless of whether they fulfilled characteristic, consistent with, or supportive classification criteria under ASTM E1588‐25. This inclusive approach was adopted to characterize the full compositional range of the particle population under thermal stress, including the bimetallic and monometallic subpopulations that become dominant at elevated temperatures. Since the sample type prevented the selection of a specific area of analysis, the analysis was performed considering a number of particles determined according to distance and temperature: 100 particles for samples at firing distances ≤50 cm and temperatures ≤750°C (including untreated samples), and 20 to 50 particles for firing distances ≥75 cm and/or temperature of 1000°C.

### 2.5 Statistical analysis

In order to investigate the effects of the experimental parameters (firing distance and exposure temperature) on metal concentrations and GSR characteristics (i.e., GSR size and morphology), statistical analyses were carried out.

Second‐degree polynomial regressions were fitted to evaluate the relationship between metal concentrations measured by ICP‐OES and the experimental parameters, firing distance (FD) and temperature (T). Specifically, separate models were fitted for Ba, Pb, Sb, Cu, and Zn concentrations, including the interaction term “firing distance × temperature” (FD × T) to assess their combined effect on metal concentrations. Model fit was evaluated using the distribution of residuals and the coefficient of determination (*R* ^2^).

Exploratory analyses were also conducted using Principal Component Analysis (PCA). For this purpose, ICP‐OES regression models and SEM‐EDS data were included in the analysis; in the latter case, the data were auto‐scaled to construct two‐dimensional plots. For SEM‐EDS, PCA was carried out considering the percentage of Pb, Ba, and Sb and particle size as quantitative variables, while firing distance, exposure temperature, morphology, and elemental composition were considered qualitative variables.

The analyses were performed using in‐house developed open‐source software in the R Studio Software environment (RStudio 2023.12.1 + 402 “Ocean Storm” Release) [25]. Statistical significance was assessed when the *p*‐value was lower than 0.05.

## 3 RESULTS

### 3.1 ICP‐OES analysis

The concentration (μg/cm^2^) of the three characteristic primer elements, lead (Pb), barium (Ba), antimony (Sb), and the two secondary elements, copper (Cu), and zinc (Zn), was determined in the untreated (U) and heat‐treated (T) samples at different temperatures (250, 750, and 1000°C) and firing distances (5, 25, 50, 75, and 100 cm) conditions. The observed values were above the method quantification limit (MQL) at all analyzed points, except for the copper measurements at 75 and 100 cm after heat treatment at 1000°C, and above the negative control samples at all firing distances at room temperature, except for the zinc concentration at 100 cm (Table S3).

Across the five different firing distances, GSR surface density decreased in both heat‐treated and untreated samples, with all conditions showing a percentage decrease of >90% from 5 to 100 cm (Figure 1). In the thermally untreated sample, lead was the most abundant element (11.22 μg/cm^2^), followed by barium (3.67 μg/cm^2^), copper (3.40 μg/cm^2^), antimony (1.47 μg/cm^2^), and zinc (0.27 μg/cm^2^) at near‐contact distance (5 cm). With increasing firing distance, the surface density of the elements gradually decreased, with lead showing values similar to those of barium and copper, whereas antimony and zinc exhibited lower surface density than the other elements (Figure 1).

**FIGURE 1:**
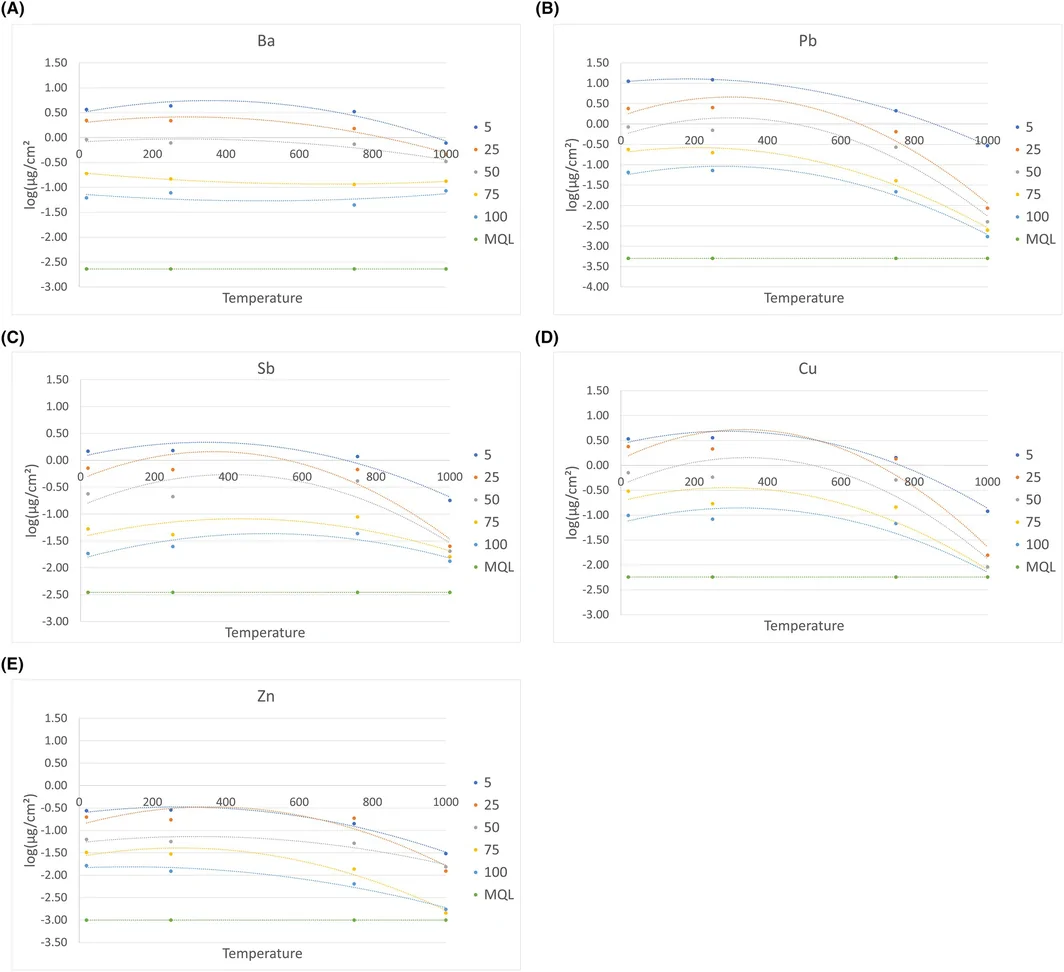
Pb, Ba, Sb, Cu, and Zn surface density on calcium silicate substrates for all barrel‐to‐target distances (x‐axis: exposure temperature, y‐axis: log(μg/cm2); MQL, method quantification limit; untreated samples were considered to be exposed to room temperature, 20°C).

Taking the temperature into account, no substantial differences were observed after exposure to 250°C compared to the untreated samples. Raising the temperature to 750°C led to a marked decrease in Pb surface density only, changing it from a relatively abundant element to one of the least prevalent, particularly over extended distances (mean percentage decrease ± SD = 74.4% ± 7.4%, min–max = 66.6%–83.0%). The surface density of the other elements, instead, did not differ substantially from those observed in the untreated samples.

At 1000°C, Pb showed a further reduction, with a mean percentage decrease of 98.6% (SD = 1.1%), followed by Cu (mean ± SD = 98.2% ± 1.5%), Zn (mean ± SD = 88.6% ± 7.9%), Sb (mean ± SD = 74.7% ± 28.0%), and Ba (mean ± SD = 42.8% ± 49.4%) (Figure 1).

These observations were corroborated by statistical analyses. Separate second‐degree polynomial regressions were fitted for Ba, Pb, Sb, Cu, and Zn surface density as a function of firing distance (FD) and temperature (T). The interaction term “firing distance × temperature” (FD × T) was also included to evaluate their combined effect on metal surface density.

ANOVA regression results showed a significant *p*‐value associated with the models (*p*‐value <0.001). The models showed residuals with normal distribution and a good fit to the experimental data (*R* ^2^ values ranging from 0.80 to 0.94), indicating a non‐linear relation between metal surface density and the experimental factors (firing distance, exposure temperature, and FD × T interaction). The summary statistics of the models are reported in Tables 1 and S4–S8.

**TABLE 1:** Summary statistics of the second‐degree polynomial regression model fitted for Ba, Pb, Sb, Cu, and Zn concentrations.

	Ba	Pb	Sb	Cu	Zn
Intercept	0.9	0.3	0.3	0.7	0.08
Firing distance (FD)	−1.4	−2.7	−0.5	−1.1	−0.09
Temperature (T)	−0.5	−1.6	−0.2	−0.6	−0.05
FD × T	0.7	2.7	0.3	0.9	0.06
FD^2^	1.0	3.1	0.4	0.6	0.04
T^2^	−0.6	ns	−0.3	ns	ns
*R* ^2^	0.928	0.801	0.904	0.941	0.897
Adjusted *R* ^2^	0.902	0.730	0.870	0.921	0.860
*F*‐statistics	35.915	11.286	26.483	45.014	24.276
(*p*‐value)	(1.67E‐07)	(1.64E‐04)	(1.14E‐06)	(3.89E‐08)	(1.95E‐06)

Abbreviations: FD, firing distance; T, exposure temperature.

Both experimental factors were inversely related to metal surface density; however, firing distance produced a steeper decline in metal surface density, approximately 2–3 times steeper than that induced by exposure temperature. The latter exerted a stronger effect on Pb surface density, as indicated by its larger (absolute) regression coefficient compared to those of the other elements.

For all the elements investigated, there was a significant antagonistic interaction between firing distance and exposure temperature (*p*‐value <0.002). Higher surface density is associated with lower temperature and shorter firing distances, and, conversely, lower metal surface density is related to longer distances and higher temperatures (Figures 2 and S3). Thus, the interaction factor is crucial for understanding data because the metal surface density at short distances and low temperatures is higher than the expected value based only on the temperature or distance.

**FIGURE 2:**
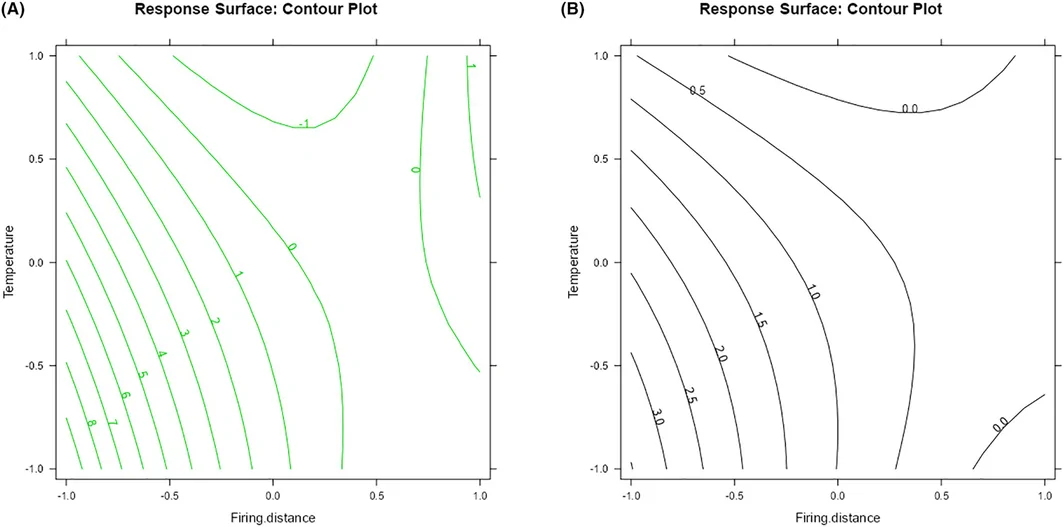
Model isoresponse plots for lead (A) and copper (B), showing the effects of the distance and temperature on metal surface density.

PCA analysis of regression models revealed that the variability in metal surface density was governed primarily by the firing distance rather than by the exposure temperature. An element‐specific pattern was also observed. In particular, Pb exhibited the strongest response to the experimental factors, showing pronounced surface density shifts associated with increasing FD × T and FD^2^. As the temperature increased, Pb surface density decreased, followed by Ba, Cu, and Sb, with Zn showing no variation (Figure 3).

**FIGURE 3:**
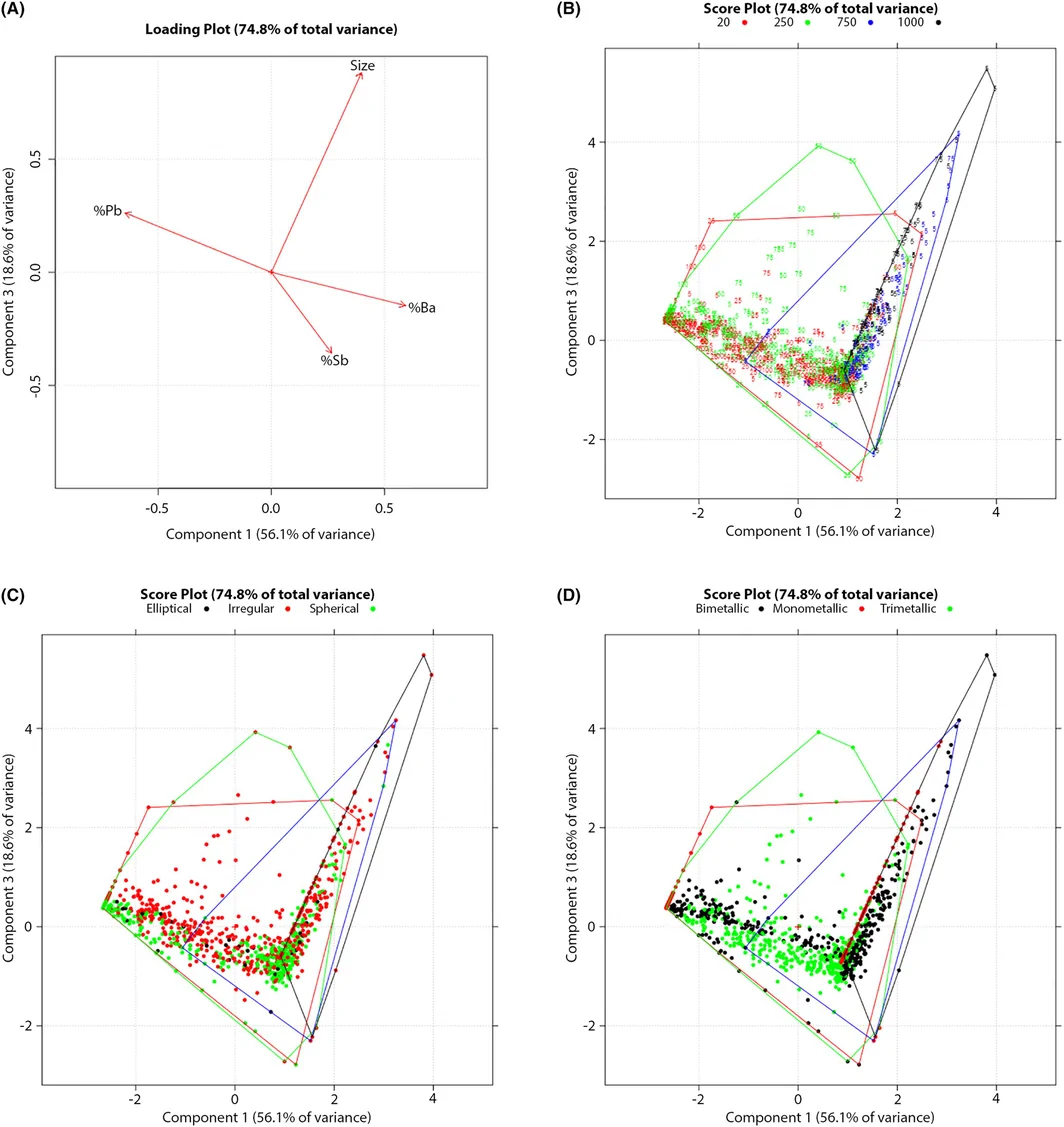
Principal Component Analysis: Loading and Score Plot of the regression models. FD, firing distance; T, exposure temperature.

Negative control samples (field blanks and absolute blanks) showed surface density below 0.02 μg/cm^2^ for all elements, excluding any possible external or environmental contamination. The reproducibility across replicate runs and the surface density gradient across distances further validates the robustness of the procedure.

### 3.2 SEM‐EDS analysis

SEM‐EDS analysis was carried out for all firing distances in both the untreated sample and the sample exposed to 250°C. For higher temperatures (750–1000°C), only two distances were selected (5 and 75 cm) to verify the behavior at limiting distances. Information was collected on about a hundred particles at shorter distances (from 5 to 50 cm) in the untreated samples and samples exposed to 250–750°C, and on fewer particles for longer barrel‐to‐target distances (75–100 cm) and/or exposure temperature of 1000°C (12–51 particles), due to the small amount of GSR deposited.

Generally, the untreated sample and the sample exposed to 250°C showed similar results. In all cases, more than 50% of the particles showed diameters included between 1 and 5 μm (min‐max: 56–87%). Most of them (about 90%) had spherical and irregular morphology, the former with slightly lower values at near‐contact ranges (5 cm) (spherical ≈ 40%, irregular = 57%). As the firing distances increased, a different distribution was observed between the two groups, depending on the thermal treatment. In the untreated sample, spherical particles initially increased at intermediate distances (25–75 cm), reaching a level approximately twice that of the irregular particles (spherical ≈ 60%, irregular ≈ 30%), before decreasing to values approaching the contact range (spherical = 41%, irregular = 53%). In the heat‐treated sample (250°C), instead, the number of irregular particles was twice that of the spherical ones at distances included between 50 and 75 cm (spherical ≈ 30%, irregular ≈ 60%). In all samples, low percentages were recorded for the elliptical morphology (lower than 13%) (Table 2).

**TABLE 2:**
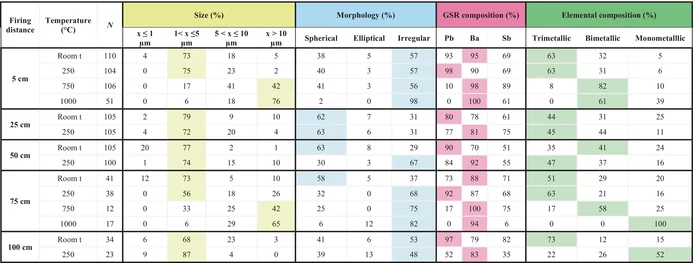
SEM‐EDS results: Percentage of particles for the variables considered (size, morphology, GSR composition, and elemental composition) under each experimental condition (firing distance and exposure temperature).

*Note*: *GSR composition* refers to the percentage of particles containing each element, whereas *elemental composition* refers to the percentage of tri‐, bi‐, and monometallic particles.

Abbreviations: *N*, number of particles analyzed; Room t, room temperature.

Concerning the chemical composition, the selected elements (Pb, Ba, Sb, Cu, Zn) were identified in all samples, although not all were present in every residue. Focusing on the three characteristic elements (Pb, Ba, and Sb), barium and lead were detected in 70–98% of the particles in both untreated and heat‐treated (250°C) samples, followed by antimony (51–82%). No peculiar distribution was observed, except for a substantial decrease in particles containing Pb (52%) and Sb (35%) in samples treated at 250°C at the longest firing distance (100 cm). 70% of the particles contained at least two characteristic elements in all samples, except for the heat‐treated sample (250°C) at 100 cm, where a higher number of particles with only a single element were detected (one element = 52%, two elements = 26%, three elements = 22%). At the other firing distances (5–75 cm), all three elements were observed in at least 35% of the particles (maximum = 73%). (Table 2).

Increasing temperature caused changes in both size and morphology. In particular, from the untreated to the heat‐treated samples, there was a progressive increase in the proportion of larger‐diameter particles (> 10 μm, from 5–10% to 65–76%), concomitant with a marked decrease in smaller‐diameter particles (1–5 μm, from 73 to 6%). A similar trend was detected in particle morphology, particularly in samples shot at 75 cm, where the proportion of irregular particles increased from 37% (untreated sample) to 82% (following treatment at 1000°C), accompanied by a concomitant decrease in the proportion of spherical particles (untreated = 58%, 1000°C = 6%). In contrast, at 5 cm, the percentages remained relatively constant up to 750°C (spherical ≈ 40%, irregular = 57%), before undergoing a drastic shift at 1000°C (spherical = 2%, irregular = 98%) (Table 2, Figure 4). At higher temperatures (≥750°C), irregular particles exhibited flattened sheath‐like structures surrounding the fibers homogeneous, in contrast to the smooth surfaces and well‐defined contours observed at lower temperatures (Figures 5 and 6).

**FIGURE 4:**
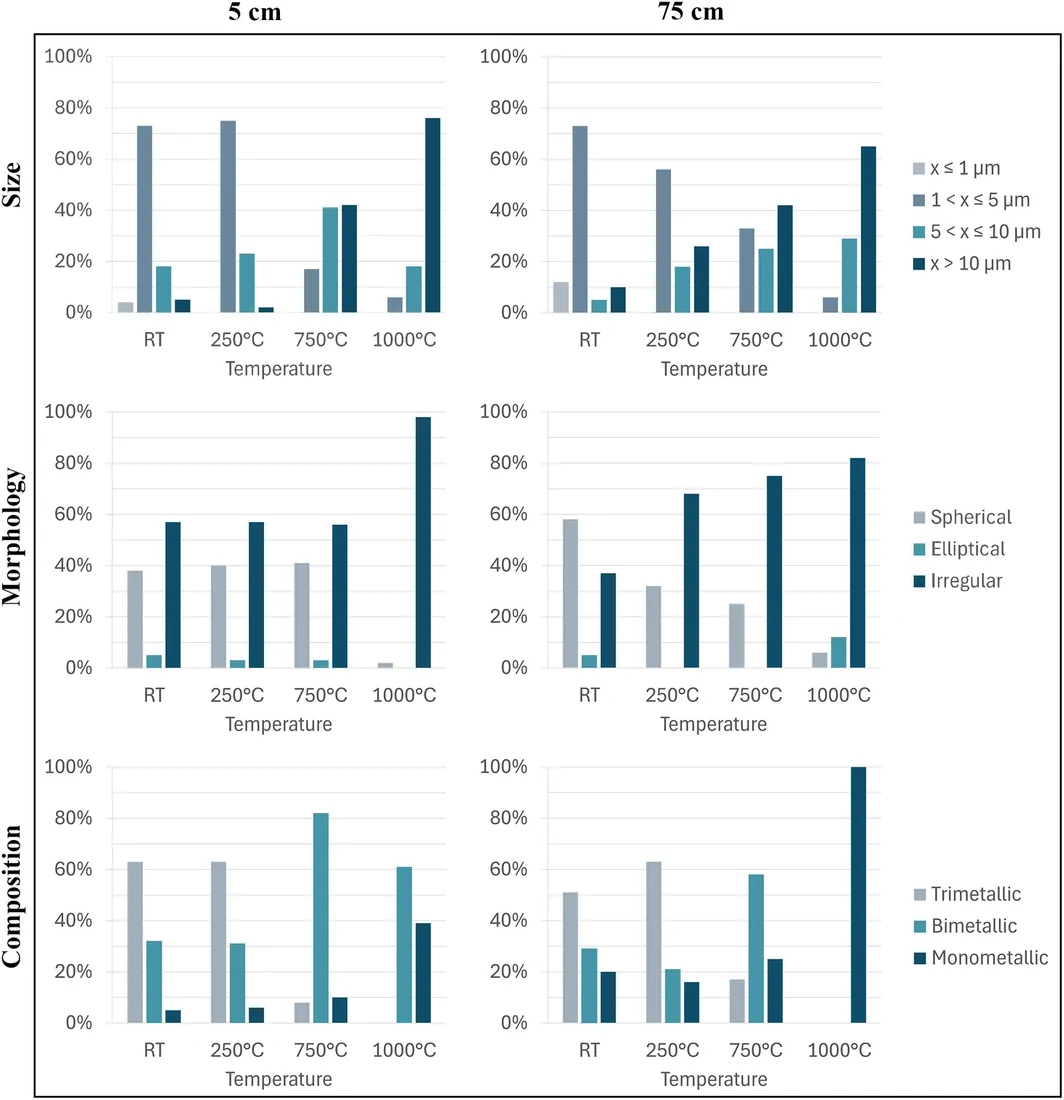
Distribution of particles for each variable (size, morphology, and composition) across the different exposure temperatures at firing distances of 5 cm and 75 cm. *Composition* refers to the percentage of tri‐, bi‐, and monometallic particles (RT: Room temperature).

**FIGURE 5:**
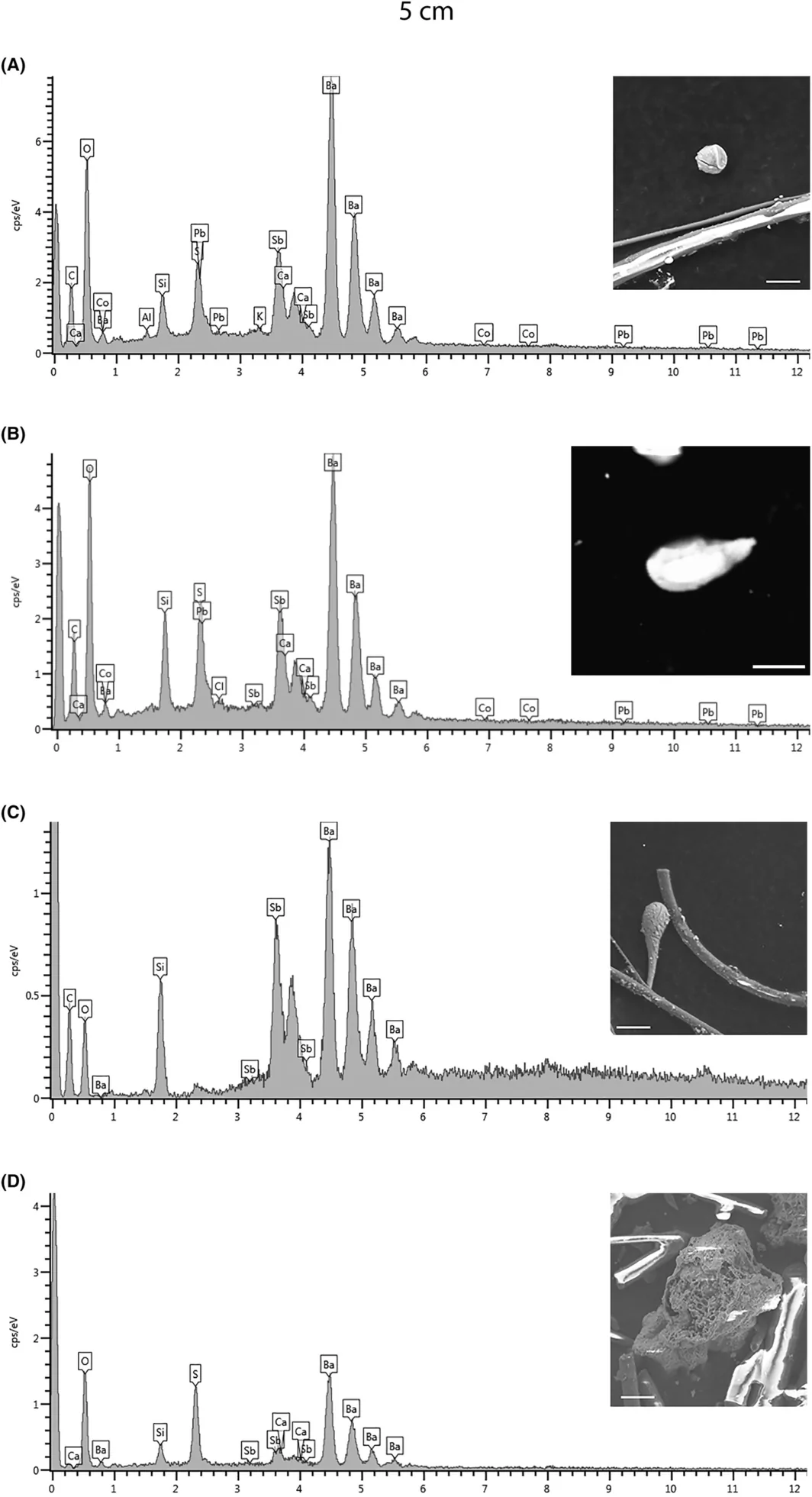
Gunshot residues collected from targets exposed to room temperature (A–C) and 1000°C (D) at a firing distance of 5 cm. The particles exhibit spherical (A), elliptical (B), and irregular (C and D) morphologies. Differences in the degree of morphological irregularity are observed between the two temperature conditions (scale bar: 10 μm).

**FIGURE 6:**
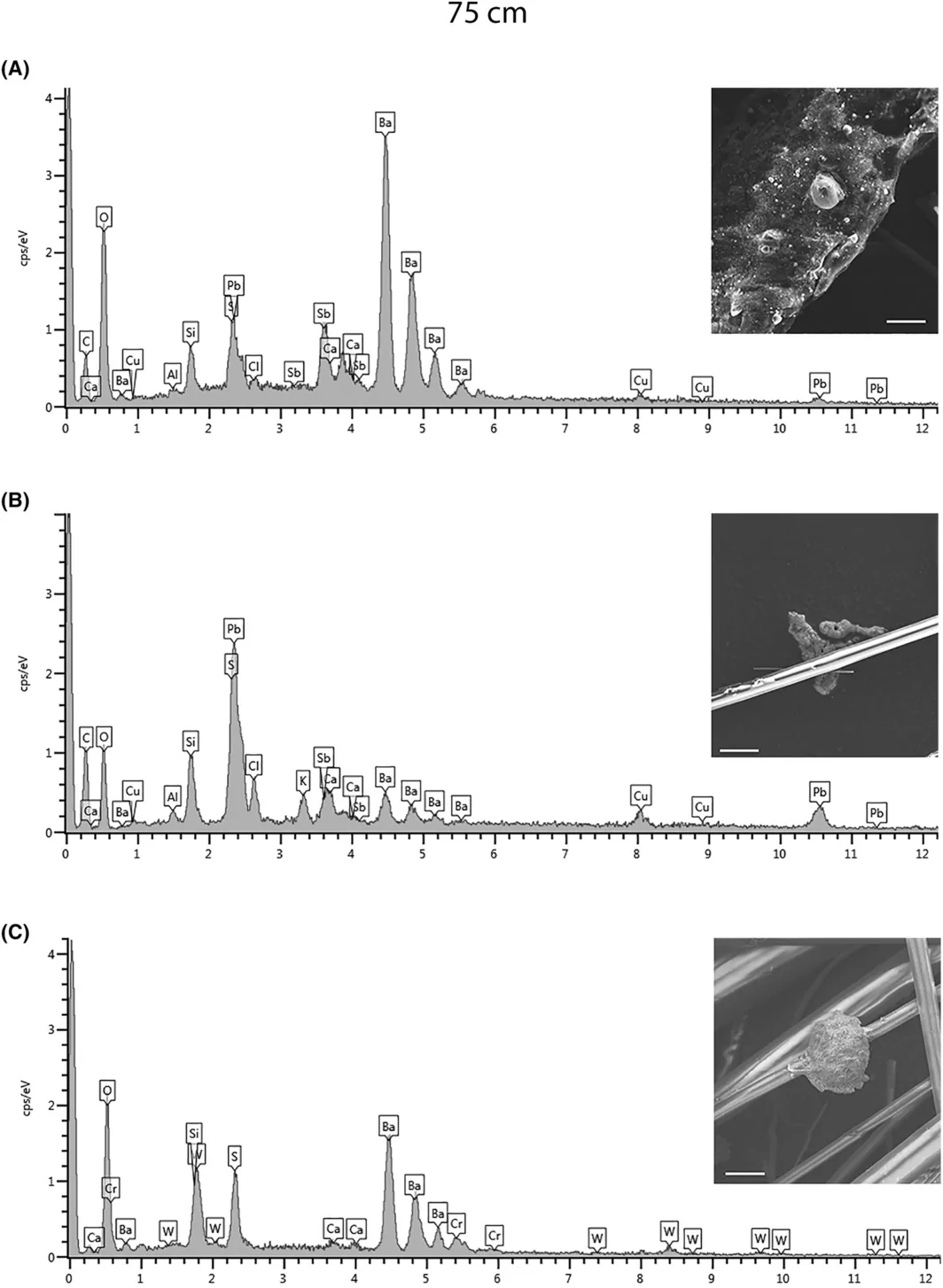
Gunshot residues collected from targets exposed to room temperature (A), 250°C (B), and 1000°C (C) at a firing distance of 75 cm. The particles exhibit spherical (A) and irregular (B and C) morphologies, with differences in the degree of morphological irregularity observed among the temperature conditions (scale bar: 10 μm).

Regarding chemical composition, the percentage of particles containing lead decreased to 10–17% after treatment at 750°C and dropped to undetectable levels after treatment at 1000°C. Barium remained the most abundant element (94–100%), followed by antimony (61–89%), although the latter dropped to 6% in samples fired at 75 cm and exposed to 1000°C. Consequently, fewer particles containing all three elements were detected (< 17% in samples treated at 750°C and undetectable in those treated at 1000°C), while the proportion of particles containing only two or a single element increased (Table 2, Figure 4). After treatment at 750°C, bimetallic particles were more abundant than monometallic ones, but their numbers decreased at 1000°C, disappearing entirely at 75 cm, where 100% of the particles were monometallic.

These observations were validated by PCA analysis (Figures 7 and S4). In Figure 7, the PCA plot accounts for 74.8% of the total variance (PC1: 56.1%; PC3: 18.6%). PC1 is mainly influenced by positive loadings for barium and antimony and a negative loading for lead, while PC3 reflects differences based on GSR size. The distribution of scores along PC1 shows a shift with increasing exposure temperatures, with GSRs collected from untreated samples and samples exposed to 250°C displaying a relatively homogeneous distribution along PC1, whereas GSRs from samples exposed to higher temperatures (750–1000°C) cluster toward higher PC1 values. The vertical spread of the scores within this cluster suggests that GSRs exposed to the highest temperatures are characterized by increasing size. The scores plot shows that most particles from the highest‐temperature samples exhibit an irregular morphology and have mainly bi‐ or monometallic composition. The convex hulls drawn around each temperature class highlight the partial overlap between groups at lower temperatures (20–250°C), while increasingly distinct separation emerges for samples acquired at higher temperatures (750–1000°C).

**FIGURE 7:**
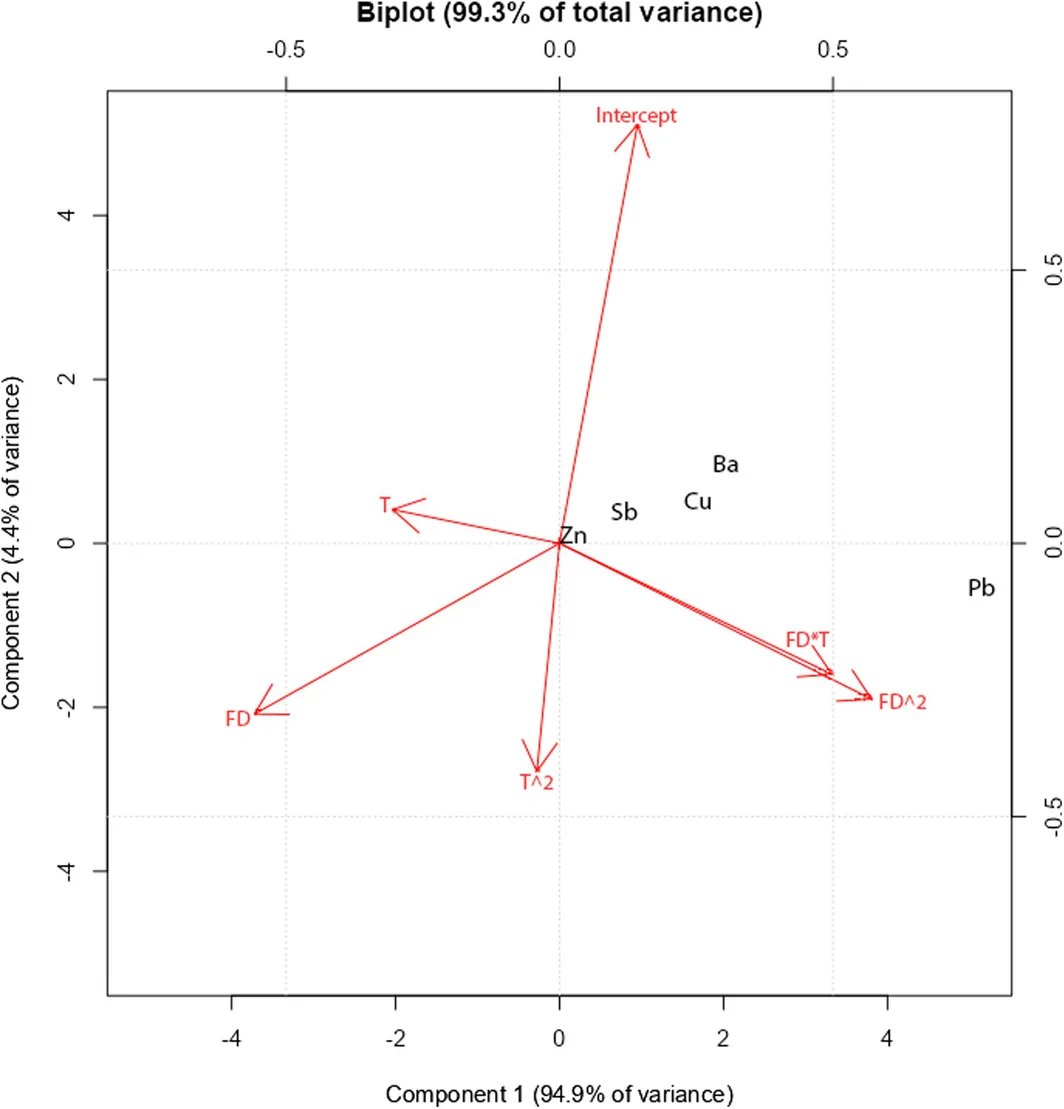
Principal Component Analysis: Loading plot (A) and score plots (B–D) of the SEM‐EDS data (percentages of Pb, Ba, and Sb and particle size) for PC1 versus PC3. Scores are colored according to (B) exposure temperature, (C) particle morphology, and (D) particle composition. In panel (B), scores are also labeled according to firing distance. Convex hulls delineate each temperature class.

## 4 DISCUSSION

The present study provides preliminary insight into the persistence and thermal modification of inorganic gunshot residue (GSR) deposited at different barrel‐to‐target distances (5–100 cm) and subsequently exposed to increasing temperatures (250–1000°C). Particles were assessed using SEM‐EDS and ICP‐OES, two techniques commonly applied in forensic GSR analysis. SEM‐EDS remains the primary method for GSR examination because it provides both morphological and elemental information, in accordance with ASTM E1588‐25 criteria [2]. ICP‐OES, while not particle‐specific, is increasingly used in forensic applications (including GSR investigations) because it offers higher quantitative sensitivity for elemental detection than SEM‐EDS [11, 19]. Some studies have already evaluated the effects of heat on GSR using SEM‐EDS and/or ICP‐based approaches [11, 16, 19]. However, these investigations were typically performed at near‐contact range and often examined a single exposure temperature, frequently using animal models (e.g., sheep limbs or bovine ribs). In contrast, the present work explored progressive thermal exposure across a broad temperature range and incorporated varying firing distances, allowing the combined influence of deposition conditions and heat treatment to be evaluated. To our knowledge, data describing the progressive alteration of GSR deposited at multiple firing distances across increasing temperatures—up to 1000°C—have not been previously reported.

### 4.1 Substrate considerations and analytical approach

The experiments were conducted on calcium silicate substrates selected for their fire‐resistant properties and their ability to retain deposited particles, thereby providing a controlled proxy for thermally altered evidence. This matrix, however, introduced practical challenges for SEM‐EDS because fiber‐rich areas interfered with a reliable assessment of particle density. To mitigate this limitation, residues were evaluated using a standardized approach based on a defined number of particles consistent with each condition, and the examination was performed across the entire stub surface. This approach supports qualitative and semi‐quantitative comparisons across conditions but does not permit robust inferences about absolute particle density on the substrate.

### 4.2 Morphological and size changes with thermal exposure

Across all experimental conditions, inorganic particles bearing GSR‐relevant elements remained detectable by SEM‐EDS. However, particles fulfilling the ASTM E1588‐25 criterion for characteristic GSR, defined by the simultaneous presence of lead, barium, and antimony, could not be observed at 1000°C at both tested distances, so that the characteristic particle population at this temperature becomes undetectable. At lower exposure temperatures (≤250°C), particle morphology was broadly consistent with that described in standard practice—often spheroidal and typically within the micrometer range (approximately 0.5–5 μm) [2]. Irregular morphologies were nevertheless frequently observed. Because particle selection for SEM‐EDS is inherently operator dependent and a density analysis was not feasible on this substrate, the apparent abundance of irregular particles may partly reflect selection effects. More generally, however, morphological variability is well documented in GSR. Non‐spherical forms (e.g., tailed, elliptical, or irregular particles) have been attributed to dynamic formation processes and collision events during cooling and transport [26, 27, 28], including impact‐related deformation reported at intermediate barrel‐to‐target distances [26]. Thermally induced changes became pronounced at ≥750°C. At these temperatures, particles exhibited progressive loss of spherical morphology and a shift toward larger features (frequently >10 μm). Importantly, the nature of “irregularity” differed with temperature: at lower temperatures, irregular particles typically displayed relatively smooth surfaces and well‐defined contours, whereas at ≥750°C particles often appeared flattened and developed sheath‐like structures associated with the substrate fibers. Similar heat‐associated alterations (including notched or indented morphologies following carbonization) have been described previously [16, 19]. Under the present conditions, these higher‐temperature morphologies are more consistent with melting, coalescence, and/or clustering of adjacent residues during heat treatment than with dynamic formation processes during the shooting event [16]. This agglomeration process has important analytical consequences. For SEM‐EDS, the apparent reduction in particle counts at elevated temperatures may partly reflect coalescence of smaller particles into fewer, larger aggregates rather than volatilization alone, complicating the use of particle counts as a quantitative proxy for GSR amount in thermally altered samples. For ICP‐OES, agglomeration is less directly relevant to the bulk elemental signal—which reflects total elemental mass rather than particle number—but may affect extraction efficiency if large, substrate‐fused aggregates are incompletely dissolved during acid digestion.

### 4.3 Elemental variation: SEM‐EDS trends, PCA, and ICP‐OES confirmation

Changes in elemental composition were supported by both SEM‐EDS and ICP‐OES. In the SEM‐EDS dataset, principal component analysis (PCA) showed that particles from samples exposed to lower temperatures (≤ 250°C) had a relatively homogeneous distribution along the axis primarily associated with composition (PC1), whereas particles from ≥750°C conditions displayed a shift toward higher PC1 scores and greater dispersion along the component most related to particle size (PC3). This pattern indicates that thermal exposure is associated with compositional variation and size changes supporting the observation that high‐temperature treatment alters the overall GSR particle population. ICP‐OES findings were consistent with these trends and with prior studies reporting reduced elemental concentrations at increasing firing distance and/or after thermal exposure [11, 13, 16, 19, 29, 30]. In earlier work, decreases have been attributed, at least in part, to volatilization of elements present in propellant and primer residues (often as oxides) when exposed to heat [16]. Because elemental composition is central to GSR interpretation (ASTM E1588‐25 [2]), characterizing how composition changes with deposition conditions and thermal history is directly relevant to casework involving burned evidence. Elemental evaluation in the present study focused on the three primary primer‐associated elements (Pb, Ba, Sb) and two additional metals (Cu and Zn), which were included to assess the persistence of common projectile/bullet‐jacket constituents under the tested conditions, as previously explored by Amadasi et al. [11, 16]. Secondary elements commonly reported in ASTM practice (e.g., Al, Ca, Si) were not evaluated because of the substrate composition and the resulting risk of confounding.

### 4.4 Multivariate modeling and differences from prior studies

A novel component of this pilot work is the application of multivariate regression modeling incorporating firing distance (FD), exposure temperature (T), their interaction (FD × T), and second‐order terms. The model identified an antagonistic interaction between FD and T for the assessed elemental profile, indicating a lesser‐than‐expected decline associated with increasing firing distance and exposure temperature. Practically, this suggests that the effect of firing distance on measured elemental concentrations may vary depending on the thermal history of the substrate.

Among the measured metals, Pb showed the greatest sensitivity to the experimental factors, followed by Ba, Cu, and Sb, whereas Zn did not show meaningful variation under the tested conditions. At first glance, the strong Pb decrease may appear inconsistent with prior studies in which Pb has often remained frequently detectable after carbonization [11, 16, 19]. This difference is plausibly explained by methodological and contextual factors: previous work generally focused on near‐contact deposition, used different substrates/animal models, and examined different ammunition/projectiles, all of which can influence baseline composition and post‐exposure persistence. The observed element‐specific behavior is also consistent with basic physicochemical considerations. Pb has the lowest melting point (327.6°C, [31]), which provides a plausible explanation for the marked reduction observed at ≥750°C. In this study, Pb transitioned from a relatively abundant element to one of the least prevalent after heating at 750°C (mean decrease 74.4%), with a further decrease after 1000°C (mean decrease 98.6%). Concordantly, SEM‐EDS showed a sharp reduction in Pb‐containing particles after 750°C (to <20%) at both tested distances (5 and 75 cm). Other elements showed pronounced reductions mainly at 1000°C, with the exception of Ba, which decreased less and remained the most frequently detected element by SEM‐EDS. This relative persistence may reflect the occurrence of Ba in more thermally stable forms (e.g., oxides), which may be less prone to volatilization under the tested conditions [32].

### 4.5 Forensic implications for classification and interpretation

The combined SEM‐EDS and ICP‐OES results help explain the observed reduction in trimetallic particles at ≥750°C. This has direct interpretative implications. Under ASTM‐based frameworks, “characteristic” GSR is typically associated with particles containing Pb, Ba, and Sb. The present findings indicate that high‐temperature exposure can reduce the proportion of particles meeting characteristic criteria, yielding particle populations more likely to be classified as “consistent with” or “commonly associated with” GSR at higher temperatures. In burned evidence, therefore, reliance on characteristic particle counts alone may increase the risk of under‐recognition or misinterpretation unless thermal effects are explicitly considered. More specifically, at 1000°C, the complete absence of trimetallic particles means that a standard ASTM‐based examination would yield a negative or inconclusive result for characteristic GSR, even in substrates known to carry IGSR. Bimetallic (Ba‐Sb) and monometallic (Ba) particles—which constitute the dominant population at this temperature—fall outside the characteristic category and would typically be classified as consistent with or supportive of GSR at best, or disregarded entirely under strict classification protocols. ICP‐OES provides quantitative data even when particle counts are insufficient for meaningful SEM‐EDS classification and its impact in firing distance determination is undoubted. Of course, its use in casework requires caution: as a bulk technique, ICP‐OES cannot resolve individual particle morphology or composition, and is therefore unable to distinguish GSR‐derived elemental signal from contributions of non‐GSR particles of environmental or occupational origin containing the same target elements (e.g., barium compounds, antimony‐based materials). In the present controlled study, the risk of contamination is mitigated by the use of process and substrate blanks and by the known GSR‐positive nature of the substrates. In practical terms, the following adaptations are recommended when evaluating GSR evidence from thermally altered remains: (I) document and report all particle types (characteristic, consistent with, and supportive) rather than applying a strict characteristic‐only filter; (II) record elemental composition in full, including bimetallic and monometallic particles, to allow post‐hoc assessment of compositional shifts consistent with thermal modification; (III) integrate SEM‐EDS and ICP‐OES data to maximize information in conditions where particle counts are limited; and (IV) include explicit reference to the thermal scenario in the interpretive statement, acknowledging the potential for under‐recognition under standard criteria.

### 4.6 Limitations and future directions

This study has several limitations. First, the calcium silicate substrate complicated SEM‐EDS evaluation by introducing fiber‐rich regions that prevented robust density assessment; nevertheless, the combined SEM‐EDS and ICP‐OES approach enabled consistent qualitative and semi‐quantitative characterization of particle size, morphology, and elemental trends across conditions. Second, the sample size was limited and only one firearm/ammunition combination was examined. Because GSR composition depends strongly on primer formulation and projectile characteristics, caution is required when extrapolating these results to other ballistic configurations. Further work should include larger sample sets, multiple ammunition types (including different primer chemistries), longer distances, additional substrates more representative of burned biological tissues, and controlled variation in exposure duration and burning conditions to better reflect the range of real‐case fire scenarios.

## 5 CONCLUSIONS

This pilot study evaluated the persistence and thermally induced modification of inorganic gunshot residue (GSR) deposited on a tissue‐like substrate at barrel‐to‐target distances of 5–100 cm and subsequently exposed to 250–1000°C. Complementary bulk elemental analysis (ICP‐OES) and particle‐based characterization (SEM‐EDS) were used to assess elemental composition, morphology, and particle size distribution. Inorganic GSR remained detectable under all experimental conditions. Notably, GSR was still observable after exposure to 1000°C, extending the thermal range examined in prior studies (to the best of our knowledge) and indicating that GSR detectability may persist after more extreme heating than previously documented.

Both firing distance and thermal exposure measurably affected GSR, but in different ways. Increasing distance primarily reduced the overall residue signal, whereas heating produced marked morphological and compositional alterations, including changes in particle size distribution and a reduction in particles meeting traditional “characteristic” criteria. Unlike much of the existing work that considers distance or heat in isolation, our data show a significant interaction between these variables, consistent with an antagonistic effect, which may further complicate interpretation in thermally damaged casework. Among the metallic elements, Pb showed the greatest thermal sensitivity, plausibly related to its low melting point, resulting in a decreased contribution of Pb‐bearing characteristic particles at higher temperatures.

These findings emphasize the need to adapt GSR interpretation strategies for burned evidence, particularly in fire‐associated deaths and gunshot injuries involving thermally altered remains. Accordingly, approaches that rely only on the traditional Pb‐Ba‐Sb triad may be less robust after intense heating than suggested by studies conducted at lower thermal exposures or on less heat‐impacted substrates. Therefore, protocols should explicitly account for thermal history and incorporate complementary criteria (e.g., morphology, particle population changes, and broader elemental profiles) when interpreting GSR in burned substrates.

## CONFLICT OF INTEREST STATEMENT

The authors declare no conflict of interest.

## Supporting information


**Figure S1:** Calcium silicate substrate targets hit at the five different barrel‐to‐target distances considered: 5, 25, 50, 75, and 100 cm (scale bar: 2 cm).
**Figure S2:** 7 × 7 cm quadrants hit at 5 and 75 cm from the barrel muzzle, untreated (Room temperature) and heat‐treated at 250°C, 750°C, and 1000°C (scale bar: 1 cm).
**Figure S3:** Model isoresponse plots for barium (A), antimony (B), and zinc (C), showing the effects of the distance and temperature on metal concentrations.
**Figure S4:** Principal Component Analysis: Loading plot (A) and score plots (B–D) of the SEM‐EDS data (percentages of Pb, Ba, and Sb and particle size) for PC1 versus PC2. Scores are colored according to (B) exposure temperature, (C) particle morphology, and (D) particle composition. In panel (B), scores are also labeled according to firing distance. Convex hulls delineate each temperature class.

**Table S1:** Calibration curves and instrumental parameters used for ICP‐OES analyses.
**Table S2:** IDL‐IQL, and MDL‐MQL for the elements of interest.
**Table S3:** Mean concentrations (and standard deviations, SD) of the selected elements on field blanks and target blanks (*n* = 4).
**Table S4:** Summary statistics of the second‐degree polynomial regression model of the Ba concentrations (SE: standard error, 95% CI: 95% confidence interval, *t* Stat: *t*‐statistic, *p*: *p*‐value).
**Table S5:** Summary statistics of the second‐degree polynomial regression model of the Pb concentrations (SE: standard error, 95% CI: 95% confidence interval, *t* Stat: *t*‐statistic, *p*: *p*‐value).
**Table S6:** Summary statistics of the second‐degree polynomial regression model of the Sb concentrations (SE: standard error, 95% CI: 95% confidence interval, *t* Stat: *t*‐statistic, *p*: *p*‐value).
**Table S7:** Summary statistics of the second‐degree polynomial regression model of the Cu concentrations (SE: standard error, 95% CI: 95% confidence interval, *t* Stat: *t*‐statistic, *p*: *p*‐value).
**Table S8:** Summary statistics of the second‐degree polynomial regression model of the Zn concentrations (SE: standard error, 95% CI: 95% confidence interval, *t* Stat: *t*‐statistic, *p*: *p*‐value).

## Data Availability

The data that supports the findings of this study are available in the supplementary material of this article.

## References

[1] Amadasi A , Borgonovo S , Brandone A , Di Giancamillo M , Cattaneo C . A comparison between digital radiography, computed tomography, and magnetic resonance in the detection of gunshot residues in burnt tissues and bone. J Forensic Sci. 2014;59(3):712–717. 10.1111/1556-4029.12304 24147424

[2] ASTM International . ASTM E1588‐25 – Standard test method for gunshot residue analysis by scanning electron microscopy/energy dispersive X‐ray spectrometry. West Conshohocken, PA: ASTM International; 2025. 10.1520/E1588-25

[3] Santos A , Ramos P , Fernandes L , Magalhães T , Almeida A , Sousa A . Firing distance estimation based on the analysis of GSR distribution on the target surface using ICP‐MS – an experimental study with a 7.65 mm × 17 mm Browning pistol (.32 ACP). Forensic Sci Int. 2015;247:62–68. 10.1016/j.forsciint.2014.12.006 25553356

[4] Wallace JS . Chemical analysis of firearms, ammunition, and gunshot residue. 2nd ed. Boca Raton FL: CRC Press; 2018.

[5] Fabbris S , Profumo A , Alberini G , Bonomi S , Milanese C , Donghi M , et al. Interaction of gunshot residues (GSR) with natural and synthetic textiles having different structural features. Talanta Open. 2020;2:100017. 10.1016/j.talo.2020.100017

[6] Merli D , Di Trocchio C , Capucciati A , Fabbris S , Profumo A , Cucca L , et al. Bullet contribution to inorganic residue on targets. Talanta Open. 2021;4:100067. 10.1016/j.talo.2021.100067

[7] Donghi M , Girella A , Pellegrino D , Maraschi F , Profumo A , Milanese C , et al. Effect of mounting a sound suppressor on distribution and total amount of inorganic gunshot residue on targets. J Forensic Sci. 2025;70(3):1165–1173. 10.1111/1556-4029.70025 PMC12046103 40178016

[8] Pope E , Juarez CA , Galloway A . Refined classification system for thermally damaged human remains by body segment. Forensic Anthropol. 2022;5(1):57–72. 10.5744/fa.2021.0008

[9] Ellingham S , Adserias‐Garriga J , Zapico SC , Ubelaker DH , editors. Burnt human remains: recovery, analysis, and interpretation. Hoboken, NJ: John Wiley & Sons; 2023.

[10] Collini F , Amadasi A , Mazzucchi A , Porta D , Regazzola VL , Garofalo P , et al. The erratic behavior of lesions in burnt bone. J Forensic Sci. 2015;60(5):1290–1294. 10.1111/1556-4029.12794 26257320

[11] Amadasi A , Merli D , Brandone A , Poppa P , Gibelli D , Cattaneo C . The survival of gunshot residues in cremated bone: an inductively coupled plasma optical emission spectrometry study. J Forensic Sci. 2013;58(4):964–966. 10.1111/1556-4029.12149 23692414

[12] Pope EJ , Smith OC . Identification of traumatic injury in burned cranial bone: an experimental approach. J Forensic Sci. 2004;49(3):431–440. 10.1520/JFS2003286 15171155

[13] Poppa P , Porta D , Gibelli D , Mazzucchi A , Brandone A , Grandi M , et al. Detection of blunt, sharp force and gunshot lesions on burnt remains: a cautionary note. Am J Forensic Med Pathol. 2011;32(3):275–279. 10.1097/PAF.0b013e3182198761 21799393

[14] Hausmann R , Betz P . Thermally induced entrance wound‐like defect of the skull. Forensic Sci Int. 2002;128(3):159–161. 10.1016/s0379-0738(02)00192-5 12175959

[15] Tsokos M . Heat‐induced post‐mortem defect of the skull simulating an exit gunshot wound of the calvarium. Forensic Sci Med Pathol. 2011;7(2):227–228. 10.1007/s12024-010-9215-4 21213070

[16] Amadasi A , Brandone A , Rizzi A , Mazzarelli D , Cattaneo C . The survival of metallic residues from gunshot wounds in cremated bone: a SEM‐EDX study. Int J Legal Med. 2012;126(4):525–531. 10.1007/s00414-011-0661-7 22249273

[17] Fais P , Giraudo C , Boscolo‐Berto R , Amagliani A , Miotto D , Feltrin G , et al. Micro‐CT features of intermediate gunshot wounds severely damaged by fire. Int J Legal Med. 2013;127(2):419–425. 10.1007/s00414-012-0775-6 23010908

[18] Amadasi A , Borgonovo S , Brandone A , Di Giancamillo M , Cattaneo C . The survival of metallic residues from gunshot wounds in cremated bone: a radiological study. Int J Legal Med. 2012;126(3):363–369. 10.1007/s00414-011-0633-y 22037901

[19] Moussa CEK , Payré B , Aries S , Grill S , Galland S , Pestourie L , et al. Effects of carbonization on gunshot residue detection in an animal model. Forensic Imaging. 2024;39:200612. 10.1016/j.fri.2024.200612

[20] Maitre M , Kirkbride KP , Horder M , Roux C , Beavis A . Current perspectives in the interpretation of gunshot residues in forensic science: a review. Forensic Sci Int. 2017;270:1–11. 10.1016/j.forsciint.2016.09.003 27889442

[21] Taudte RV , Beavis A , Blanes L , Cole N , Doble P , Roux C . Detection of gunshot residues using mass spectrometry. Biomed Res Int. 2014;2014:965403. 10.1155/2014/965403 PMC4055073 24977168

[22] Brede U , Hagel R , Redecker KH , Weuter W . Primer compositions in the course of time: from black powder and SINOXID to SINTOX compositions and SINCO booster. Propellants, Explos, Pyrotech. 1996;21:113–117. 10.1002/prep.19960210302

[23] De Souza AM , Silva R , Rodrigues MA , Rosa ID , Do Prado CB , Gurgel MM . Statistical analysis of GSR particles morphometry using the CART method. Perspect Legal Forensic Sci. 2025;2(2):10011. 10.70322/plfs.2025.10011

[24] Scientific Working Group for Gunshot Residue (SWGGSR) . Guide for primer gunshot residue analysis by scanning electron microscopy/energy dispersive X‐ray spectrometry. 2011. Available from: https://www.nist.gov/system/files/documents/2024/08/30/SWGGSR‐Guide‐for‐Primer‐GSR‐Analysis‐by‐SEM‐Xray.pdf. Accessed 18 Jul 2026.

[25] RStudio Team . RStudio: Integrated development environment for R. Boston, MA: RStudio, PBC; 2023. Available from: https://www.rstudio.com. Accessed 18 Jul 2026

[26] Burnett B . The form of gunshot residue is modified by target impact. J Forensic Sci. 1989;34(4):808–822. 10.1520/JFS12709J

[27] Brożek‐Mucha Z . Chemical and morphological study of gunshot residue persisting on the shooter by means of scanning electron microscopy and energy dispersive X‐ray spectrometry. Microsc Microanal. 2011;17(6):972–982. 10.1017/S1431927611012141 22051052

[28] Kara l , Sarikavak Y , Lisesivdin SB , Kasap M . Evaluation of morphological and chemical differences of gunshot residues in different ammunitions using SEM/EDS technique. Environ Forensic. 2016;17(1):68–79. 10.1080/15275922.2015.1133729

[29] Turillazzi E , Di Peri GP , Nieddu A , Bello S , Monaci F , Neri M , et al. Analytical and quantitative concentration of gunshot residues (Pb, Sb, Ba) to estimate entrance hole and shooting‐distance using confocal laser microscopy and inductively coupled plasma atomic emission spectrometer analysis: an experimental study. Forensic Sci Int. 2013;231(1–3):142–149. 10.1016/j.forsciint.2013.04.006 23890629

[30] Amadasi A , Gibelli D , Mazzarelli D , Porta D , Gaudio D , Salsarola D , et al. Assets and pitfalls of chemical and microscopic analyses on gunshot residues in skeletonized bodies: a report of five cases. Int J Legal Med. 2015;129(4):819–824. 10.1007/s00414-014-1107-9 25394744

[31] Angstrom Sciences . Melting points of elements reference table. Available from: https://angstromsciences.com/melting‐points‐of‐elements‐reference. Accessed 16 Jun 2025

[32] Dalby O , Butler D , Birkett JW . Analysis of gunshot residue and associated materials: a review. J Forensic Sci. 2010;55(4):924–943. 10.1111/j.1556-4029.2010.01370.x 20384934

