# Supplementary material for: Thermal persistence and physicochemical modification of inorganic gunshot residue (IGSR)
Source: J Forensic Sci. 2026 Aug 10;71(6):2670–84. doi: 10.1111/1556-4029.70434 (PMC13736404; doi:10.1111/1556-4029.70434)
Supplement: Supplementary file 1 [file JFO-71-2670-s001.docx]

**Supporting Information**

FIGURE S1	Calcium-silicate substrate targets hit at the five different barrel-to-target distances considered: 5 cm, 25 cm, 50 cm, 75 cm, and 100 cm (scale bar: 2 cm).

FIGURE S2	7 x 7 cm quadrants hit at 5 cm and 75 cm from the barrel muzzle, untreated (Room temperature) and heat-treated at 250°C, 750°C, and 1000°C (scale bar: 1 cm).

FIGURE S3	Model isoresponse plots for barium (a.), antimony (b.), and zinc (c.), showing the effects of the distance and temperature on metal concentrations.

FIGURE S4	Principal Component Analysis: Loading plot (a) and score plots (b–d) of the SEM–EDS data (percentages of Pb, Ba, and Sb and particle size) for PC1 *versus* PC2. Scores are coloured according to (b) exposure temperature, (c) particle morphology, and (d) particle composition. In panel (b), scores are also labelled according to firing distance. Convex hulls delineate each temperature class.
