# Supplementary material for: Thermal persistence and physicochemical modification of inorganic gunshot residue (IGSR)
Source: J Forensic Sci. 2026 Aug 10;71(6):2670–84. doi: 10.1111/1556-4029.70434 (PMC13736404; doi:10.1111/1556-4029.70434)
Supplement: Supplementary file 2 [file JFO-71-2670-s002.docx]

**Supporting Information**

TABLE S1	Calibration curves and instrumental parameters used for ICP-OES analyses.

| **Element** | **Calibration curve** | **R2** | **Wavelength**  **(nm)** | **Lecture mode** |
| --- | --- | --- | --- | --- |
| **Pb** | Signal (cps) = 3502.1 x Concentration (mg/L) | 0.9985 | 220.353 | Axial |
| **Ba** | Signal (cps) = 60902.1 x Concentration (mg/L) | 0.9993 | 455.403 | Radial |
| **Sb** | Signal (cps) = 1910.3 x Concentration (mg/L) | 1.0000 | 206.833 | Axial |
| **Cu** | Signal (cps) = 48093.6 x Concentration (mg/L) | 0.9989 | 324.754 | Axial |
| **Zn** | Signal (cps) = 23035.4 x Concentration (mg/L) | 0.9999 | 213.856 | Axial |

TABLE S2	IDL-IQL, and MDL-MQL for the elements of interest.

| **Element** | **IDL**  **(mg/L)** | **IQL**  **(mg/L)** | **MDL**  **(μg/cm^2^)** | **MQL**  **(μg/cm^2^)** |
| --- | --- | --- | --- | --- |
| **Pb** | 0.0004 | 0.001 | 0.0002 | 0.0005 |
| **Ba** | 0.002 | 0.005 | 0.0013 | 0.0023 |
| **Sb** | 0.004 | 0.007 | 0.0021 | 0.0035 |
| **Cu** | 0.008 | 0.011 | 0.004 | 0.0057 |
| **Zn** | 0.001 | 0.002 | 0.0007 | 0.001 |

TABLE S3	Mean concentrations (and standard deviations, SD) of the selected elements on field blanks and target blanks (n=4).

|  | Pb | Ba | Sb | Cu | Zn |
| --- | --- | --- | --- | --- | --- |
| Field blank (μg/cm^2^)  Mean (SD) | 0.0045  (0.0006) | 0.0069  (0.0021) | 0.0013  (0.0003) | 0.0052  (0.0018) | 0.0196  (0.0028) |
| Target blank (μg/cm^2^)  Mean (SD) | 0.00041  (0.00017) | 0.0047  (0.0002) | 0.00084  (0.00012) | 0.0028  (0.0002) | 0.0093  (0.0055) |

TABLE S4	Summary statistics of the second-degree polynomial regression model of the Ba concentrations (SE: standard error, 95% CI: 95% confidence interval, t Stat: t-statistic, p: p-value).

**Barium**

| Parameter | Coefficient | SE | 95% CI | t Stat | p |
| --- | --- | --- | --- | --- | --- |
| (Intercept) | 0.9 | 0.2 | (0.5, 1.4) | 4.268 | 0.000781 |
| Firing distance (FD) | -1.4 | 0.1 | (-1.7, -1.1) | -10.917 | 3.12E-08 |
| Temperature (T) | -0.5 | 0.1 | (-0.7, -0.2) | -4.011 | 0.00129 |
| FD*T | 0.7 | 0.2 | (0.3, 1.0) | 4.198 | 0.000894 |
| FD^2^ | 1.0 | 0.2 | (0.5, 1.5) | 4.259 | 0.000794 |
| T^2^ | -0.6 | 0.3 | (-1.1, -0.05) | -2.335 | 0.0350 |

R^2^: 0.928; adjusted R^2^: 0.902

F-statistics: 35.915 on 5 and 14 df, p-value: 1.67e-07

TABLE S5	Summary statistics of the second-degree polynomial regression model of the Pb concentrations (SE: standard error, 95% CI: 95% confidence interval, t Stat: t-statistic, p: p-value).

**Lead**

| Parameter | Coefficient | SE | 95% CI | t Stat | p |
| --- | --- | --- | --- | --- | --- |
| (Intercept) | 0.3 | 1.0 | (-1.8, 2.3) | 0.286 | 0.779 |
| Firing distance (FD) | -2.7 | 0.6 | (-3.9, -1.4) | -4.672 | 0.00036 |
| Temperature (T) | -1.6 | 0.5 | (-2.7, -0.5) | -3.060 | 0.00848 |
| FD*T | 2.7 | 0.7 | (1.1, 4.2) | 3.704 | 0.00236 |
| FD^2^ | 3.1 | 1.0 | (1.0, 5.2) | 3.122 | 0.0075 |
| T^2^ | -0.4 | 1.1 | (-2.8, 1.9) | -0.384 | 0.707 |

R^2^: 0.801; adjusted R^2^: 0.730

F-statistics: 11.286 on 5 and 14 df, p-value: 1.64e-04

TABLE S6	Summary statistics of the second-degree polynomial regression model of the Sb concentrations (SE: standard error, 95% CI: 95% confidence interval, t Stat: t-statistic, p: p-value).

**Antimony**

| Parameter | Coefficient | SE | 95% CI | t Stat | p |
| --- | --- | --- | --- | --- | --- |
| (Intercept) | 0.3 | 0.09 | (0.1, 0.5) | 3.741 | 0.00219 |
| Firing distance (FD) | -0.5 | 0.06 | (-0.6, -0.4) | -8.946 | 3.64E-07 |
| Temperature (T) | -0.2 | 0.05 | (-0.3, -0.07) | -3.474 | 0.00372 |
| FD*T | 0.3 | 0.07 | (0.1, 0.4) | 3.998 | 0.00132 |
| FD^2^ | 0.4 | 0.1 | (0.2, 0.6) | 3.869 | 0.00170 |
| T^2^ | -0.3 | 0.1 | (-0.5, -0.05) | -2.628 | 0.0199 |

R^2^: 0.904; adjusted R^2^: 0.870

F-statistics: 26.483 on 5 and 14 df, p-value: 1.14e-06

TABLE S7	Summary statistics of the second-degree polynomial regression model of the Cu concentrations (SE: standard error, 95% CI: 95% confidence interval, t Stat: t-statistic, p: p-value).

**Copper**

| Parameter | Coefficient | SE | 95% CI | t Stat | p |
| --- | --- | --- | --- | --- | --- |
| (Intercept) | 0.7 | 0.2 | (0.4, 1.1) | 4.385 | 0.000623 |
| Firing distance (FD) | -1.1 | 0.1 | (-1.3, -0.8) | -10.451 | 5.4E-08 |
| Temperature (T) | -0.6 | 0.09 | (-0.8, -0.4) | -6.939 | 6.88E-06 |
| FD*T | 0.9 | 0.1 | (0.6, 1.1) | 6.705 | 1E-05 |
| FD^2^ | 0.6 | 0.2 | (0.2, 1.0) | 3.528 | 0.00335 |
| T^2^ | -0.4 | 0.2 | (-0.8, 0.03) | -1.994 | 0.0660 |

R^2^: 0.941; adjusted R^2^: 0.921

F-statistics: 45.014 on 5 and 14 df, p-value: 3.89e-08

TABLE S8	Summary statistics of the second-degree polynomial regression model of the Zn concentrations (SE: standard error, 95% CI: 95% confidence interval, t Stat: t-statistic, p: p-value).

**Zinc**

| Parameter | Coefficient | SE | 95% CI | t Stat | p |
| --- | --- | --- | --- | --- | --- |
| (Intercept) | 0.08 | 0.02 | (0.04, 0.1) | 4.401 | 0.000604 |
| Firing distance (FD) | -0.09 | 0.01 | (-0.1, -0.07) | -8.372 | 8.04E-07 |
| Temperature (T) | -0.05 | 0.01 | (-0.07, -0.02) | -4.668 | 0.000363 |
| FD*T | 0.06 | 0.01 | (0.03, 0.09) | 4.146 | 0.000989 |
| FD^2^ | 0.04 | 0.02 | (0.004, 0.09) | 2.346 | 0.0342 |
| T^2^ | -0.04 | 0.02 | (-0.09, 0.003) | -2.007 | 0.0644 |

R^2^: 0.897; adjusted R^2^: 0.860

F-statistics: 24.276 on 5 and 14 df, p-value: 1.95e-06
